# A high-density genetic map and growth related QTL mapping in bighead carp (*Hypophthalmichthys nobilis*)

**DOI:** 10.1038/srep28679

**Published:** 2016-06-27

**Authors:** Beide Fu, Haiyang Liu, Xiaomu Yu, Jingou Tong

**Affiliations:** 1 State Key Laboratory of Freshwater Ecology and Biotechnology, Institute of Hydrobiology, Chinese Academy of Sciences, Wuhan 430072,China; 2 University of Chinese Academy of Sciences, Beijing 100039, China

## Abstract

Growth related traits in fish are controlled by quantitative trait loci (QTL), but no QTL for growth have been detected in bighead carp (*Hypophthalmichthys nobilis*) due to the lack of high-density genetic map. In this study, an ultra-high density genetic map was constructed with 3,121 SNP markers by sequencing 117 individuals in a F_1_ family using 2b-RAD technology. The total length of the map was 2341.27 cM, with an average marker interval of 0.75 cM. A high level of genomic synteny between our map and zebrafish was detected. Based on this genetic map, one genome-wide significant and 37 suggestive QTL for five growth-related traits were identified in 6 linkage groups (i.e. LG3, LG11, LG15, LG18, LG19, LG22). The phenotypic variance explained (PVE) by these QTL varied from 15.4% to 38.2%. Marker within the significant QTL region was surrounded by *CRP1* and *CRP2*, which played an important role in muscle cell division. These high-density map and QTL information provided a solid base for QTL fine mapping and comparative genomics in bighead carp.

High resolution linkage maps have become indispensable to many genetic studies, such as molecular marker-assisted selection (MAS)^1^, genome scaffolding and assembly^2^, comparative genomic analysis^3^ and fine-scale quantitative trait locus (QTL) mapping^4^. Large numbers of genetic markers available is a key prerequisite for constructing a high-resolution linkage map, especially single nucleotide polymorphisms (SNPs) markers, which also played an important role in ecological, genetics and evolutionary studies^5^,^6^,^7^,^8^. Next generation sequencing opened up new possibilities in SNP marker’s developing and has made it in a rapid and cost-effective manner^6^. Recent years, many genotyping-by-sequencing (GBS) methods which combined restriction enzymes and high-throughput next generation sequencing have been developed^9^,^10^,^11^,^12^ and used in aquaculture species^12^,^13^,^14^,^15^. In particular, restriction-sites associated DNA sequencing (RAD-seq) is convenient for non-model species^16^,^17^. Through the massively parallel sequencing of DNA sequences flanking restricted sites, RAD-seq identifies hundreds or thousands SNPs simultaneously for multiple samples. But there are a lot of pitfalls in RAD-seq, such as complicated manual operations and uneven genome coverage. Then, methods with simpler library preparation protocols were developed. For example, 2b-RAD method developed by Wang *et al*.^10^ has many benefits compared with other methods, such as easy to handle, less time needed for manual operation and even distribution on genome.

Bighead carp (*Hypophthalmichthys nobilis*) belongs to family of Cyprinidae. This species has 24 pairs of chromosomes and the genome size is about 0.9 Gb^18^. The annual production of bighead carp reached 3.25 million tons worldwide in 2014, and over 98% of the production was from China (FAO, 2014). Bighead carp is one of most important and valuable aquaculture species in China, with annual production rank the fourth among all freshwater species in 2012^19^. Although considerable work had been done for bighead carp to increase production, some specific characters hindered achievement of the traditional selective breeding based on phenotypes, such as very long breeding cycle (i.e. 4 years for male and 4–5 years for female), lack of large-scale genomic resource and short of markers tightly associated with growth rate. These disadvantages made it infeasible to select phenotypes for strains or varieties with higher growth rate in a short time. MAS can overcome those disadvantages in traditional selection by using DNA markers tightly linked to some QTL to help phenotypic screening^20^,^21^. This method can increase the accuracy and efficiency of selection, thereafter is an ideal mean for traits that are difficult to be improved by traditional selection.

In bighead carp, some work had been done on development of co-dominate DNA markers and linkage map construction^22^,^23^,^24^. However, the resolution of these maps isn’t high enough to detect QTL. The reason for that is genotyping large numbers of simple sequence repeats (SSRs) needs a lot of time and human labor. Now, with the development of sequencing, rapid genotyping of tens of thousands of SNP markers is accessible, which makes construction of a high-density SNP genetic map easy to come^6^.

The main purpose of our present study is to construct a high-density linkage map with only SNP markers to facilitate QTL mapping for growth related traits, such as body weight (BW), body length (BL), total length (TL), head length (HL) and body height (BH). Here, we constructed a linkage map with 3,121 markers using 2b-RAD on Illumina HiSeq 2500 platform. A major QTL for body length were detected on linkage group (LG) 15. Besides, 37 suggestive QTL were detected for five growth-related traits, whose phenotypic variation explained (PVE) varied from 15.4% to 38.2%. The genetic map and QTL can be used in breeding populations for genetic improvement of bighead carp.

## Results

### 2b-RAD Sequencing

All sequencing libraries for the full-sibling family were built with modified 2b-RAD methods^10^. After filtering low quality reads, the sequencing depth for the two parents was 4,852,376 and 5,162,352 reads, respectively. While the average depth for all progenies was 2.128 M reads per sample and total reads number for them was 249,204,191 reads (Supplementary Table S1). A total of 70,504 loci were detected after reads from parents clustering. The average depths for all the loci were 70.98-fold for parents and 30.17-fold for progenies. Finally, we mapped all the reads from parents and offspring to the loci and genotyped every locus with at least 80% of progenies presented. We found 7,786 loci were polymorphic and used them in the following map construction. Among these loci, 5,567 were co-dominant loci which were present in both parents. While the rest 2,219 were dominant loci which were present only in one of the parents.

### High-resolution linkage mapping

**Figure 1:**
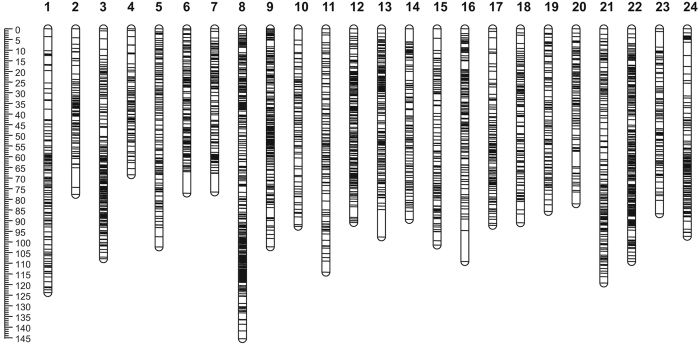
Genetic lengths and marker distribution of 24 linkage groups in the sex-averaged linkage map of bighead carp. A black bar indicated a 2b-RAD marker. The scaleplate on the left indicated genetic distance (centiMorgan as unit).

After discarding 4,463 SNPs with segregation distortion the rest 3,323 markers were used in sex-average linkage group assignment . Finally, 3,121 markers were placed onto 24 linkage groups (LGs) with a LOD (logarithm of odds threshold) of 11.0, designated as LG1-LG24, corresponding to the LG names of map constructed by micro-satellite markers^23^ (Fig. 1). The types of all 3,121 markers were illustrated in Supplementary Figure 2. The total length of the map was determined to be 2341.27 cM, with an average interval of 0.75 cM. About 97.4% of all markers’ interval was within 3 cM and 75.2% of them shorter than 1 cM. The sizes of linkage groups range from 68.53 cM to 145.42 cM (Table 1). For markers in each LG, map length calculations were performed based on recombination frequencies. The information of all markers on the LGs is presented in Supplementary Table S2, which includes marker name, sequence, and genetic position on each LG. The largest gap in the map was 14.55 cM in LG16, followed by 12.86 cM in LG13. Though we removed all skewed markers with P < 0.001, we found 184 skewed markers with P < 0.05 and 257 with P < 0.01 placed on the final map. An area containing four skewed markers was defined as a segregation distortion region (SDR). Four SDRs were detected in the map, and two of them were in LG13, which were separated by only one un-skewed marker. The other two were in LG21 and LG23. These SDRs may be related with preferential selection and would not affect the accuracy of the genetic map^25^,^26^. We mapped all 16 skewed markers to bighead carp genome (unpublished data) and got only three genes with define functions, *abi1a*, *dtx4* and *b4galt2*.

**Figure 2:**
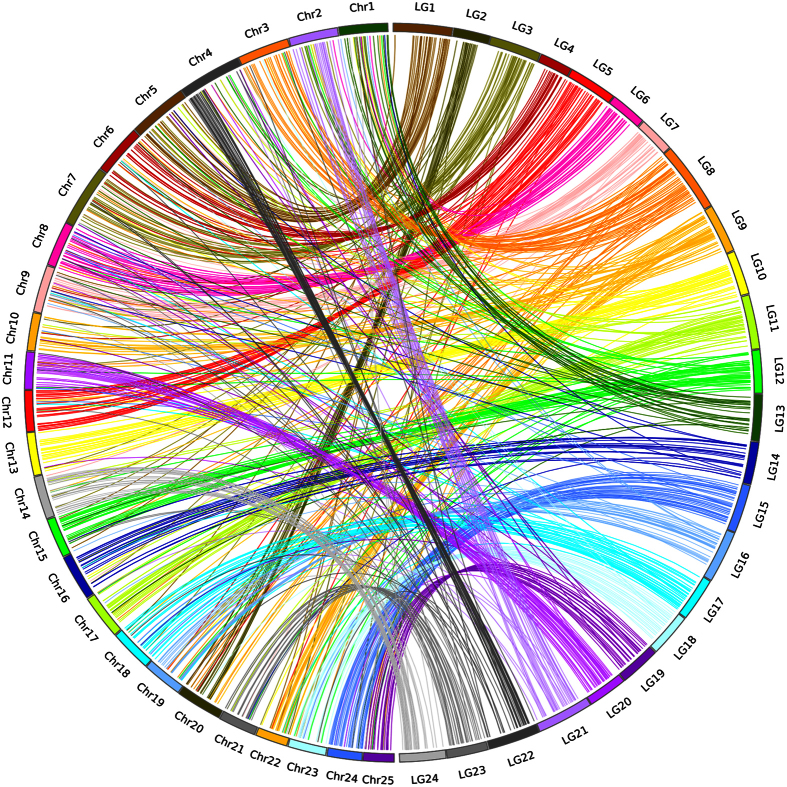
Circos mapping of 24 linkage group of bighead carp (right semi-cirle) to the 25 linkage group of zebrafish (left semi-circle).

To uncover the macro-collinearity between bighead carp and zebrafish, we mapped all 3,121 markers on final map to zebrafish genome and got 873 markers were located on 25 chromosomes of zebrafish (Supplementary Table S5). 23 pairs of 1 to 1 synteny on chromosome level were uncovered, except for LG 9 in bighead carp was mapped to Chr10 and Chr22 in zebrafish (Fig. 2). Within each pair of chromosomes, the map position of markers also showed good synteny with their corresponding loci in zebrafish genome.

### QTL mapping of growth-related traits

Pairwise comparisons among five growth-related traits (TL, BL, HL, BW, BH) using Pearson’s correlation revealed that all of them were highly correlated (P < 0.01) (Table 2). The highest correlation coefficients were 0.98 between TL and BL, and the lowest correlation coefficients were 0.82 between BH and HL.

**Figure 3:**
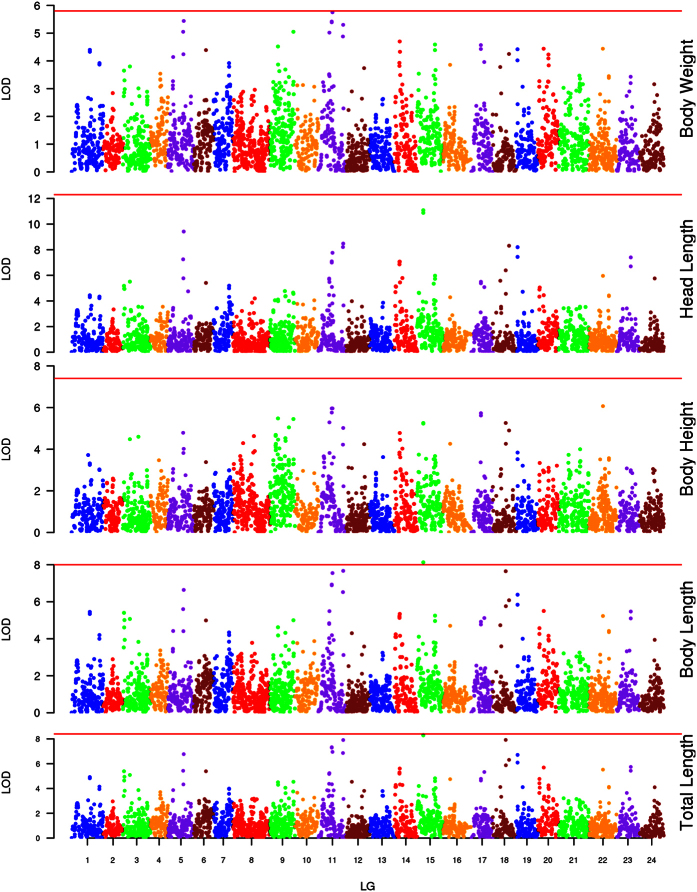
LOD scores along the 24 linkage groups for variation in 5 growth-related traits in bighead carp. The red horizontal line on each trait indicates the LOD for genome-wide significances for that trait.

We used the phenotype data above to detect QTL affecting growth-related traits and found they were located on various linkage groups in linkage-group-wide scale (Table 3 and Fig. 3). TL had 9 QTL, and *qTL15-a,* the most prominent QTL on LG15, accounted for 30.2% of the PVE. Another two QTL on LG 11 within a region of 3 cM (position of 52.31 cM and 55.22 cM) accounted for 27.3% and 26.1% of PVE. The genetic effects of other QTL were comparatively smaller. Nine QTL for BL were identified, all of which were the same as that for TL. The PVE of these nine QTL varied form 18.2–29.5%. We also found 9 QTL for BH, the largest of which is *qBH22-a* on LG22 with 23.2% of the PVE. For HL, only 4 QTL were found, with the largest effect displayed by *qHL15-a* on LG15, explaining 38.3% of the PVE. Seven QTL were found for BW, and the largest effect was on *qBW11-b*, explaining 22.1% of the PVE.

**Figure 4:**
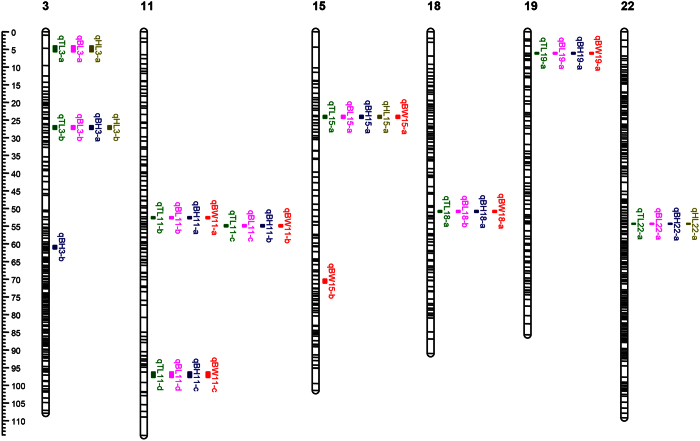
Genomic QTL distribution on 6 different chromosomes.

However, in these 38 QTL, only one QTL was of the genome-wide level of significance. After counting the frequency of the QTL in the five traits, we found 11 different loci on the genome (Fig. 4). Within which, one co-localization QTL (*qBW15-a*) on LG15 was common to all five traits, while 7 other co-localization QTL were common to four traits, and the rest 3 QTL were associated with 3 and 1 traits, respectively (Supplementary Table S3). After mapping 11 markers in these QTL to bighead carp genome (unpublished data), we found 12 genes closing to the mapping position. Then these genes were blasted to the protein database of zebrafish and got the corresponding protein. Co-localization QTL for five traits (Marker: *ref-8593_27*) were surrounded by *CRP1* and *CRP2*, which had good synteny compared with zebrafish genome. The closest gene next to co-localization QTL for four traits (Marker: *ref-67070_2*) was *SASH1*, which regulates cell proliferation and apoptosis in human^27^,^28^.

## Discussion

In this work, we constructed a high-density genetic map for bighead carp via 2b-RAD and used it to detect growth-related QTL. Linkage map is an important pre-requisite for mapping QTL of interested traits for a given species. In bighead carp, two genetic maps have been published previously: one was constructed by SSR and AFLP markers^22^, the other was constructed solely by SSR markers^23^. Table 4 summarized the main differences in the parameters derived from them. For the first one, 120 co-dominate markers were used and resulted in 39 LG, which is much more than the number of haploid chromosomes in bighead carp (N = 24)^29^. The second generation genetic map made great improvement, which involved 659 co-dominate markers and formed exactly 24 LG. And the map resolution also narrowed from 3.9 cM to 2.9 cM and the map length doubled from 852.0 cM to 1,917.3 cM^23^. Although the density and length of linkage map had been improved in the last few years, it’s still far from satisfaction for genetic studies and MAS in bighead carp. The emergency and development of next generation sequencing (NGS) technology has mad RAD-seq a feasible way to genotyping the whole genome in a short time. However, library construction for RAD-seq is a labor-intensive and time-consuming work. Therefore, a lot of modifications had been proposed, such as ddRAD^30^, SLAF^12^,^31^ and 2b-RAD^10^. 2b-RAD provided a streamlined alternative to existing RAD-seq library construction method; for it can be done in 4 hours with all reactions happened consecutively in a single well. At the same time, 2b-RAD can detected almost every restriction site in the genome in parallel whereas other RAD-seq methods can only get a subset of total sites due to the multiple times’ size selection^9^,^10^. The third advantage that 2b-RAD provided is its flexible choice of selective adaptor, which can adjust the marker density in the genome. This choice can balance level of genotyping detail against sequencing throughput depending on various types of study^13^.

In this study, we used a crossing strategy of a male and a female bighead carp and their 117 progenies as mapping population. Then genotyping SNPs for the population with 2b-RAD and got a high-density linkage map of 3,121 markers. This number is almost five times of that in previous linkage map constructed by SSR markers, and the average distance between markers dropped drastically from 2.9 cM to 0.75 cM. With more markers used in our map, the observed genome length was 2341.27 cM for the consensus map, which is 22% longer than previous map constructed by SSRs^23^. The synteny analysis between this genetic map and zebrafish genome showed 23 pairs of ortholog chromosomes, except for the LG9 in bighead carp corresponding to Chr10 and Chr22 in zebrafish genome. This result of a good macro-collinearity is consistent with previous study on comparative analysis of bighead carp genome and zebrafish genome^32^ Meanwhile, the macro-collinearity above justified the LOD used in this study is adequate in high-density SNP genetic map construction^33^, although they are much higher compared with those LOD value used in SSR genetic map^23^,^34^.

To increase the genomic coverage on the final genetic map, skewed markers were also used in the map construction. In total, 16 skewed markers in four segregation distortion regions were found. The annotation of these markers found three genes with known function, *abi1a*, *dtx4* and *b4galt2*. Gene *abi1* play a role in the progression of several malignancies including colon cancer^35^, ovarian cancer^36^ and breast cancer^37^. Gene *b4galt2* act as a target gene of p53-mediated HeLa cell apoptosis^38^. Marker distortion might be caused by special selection on some type of genotype, such as double recessive of some lethal genes, or caused by zygotic selection^39^. It has been reported that skewed markers can be beneficial to map construction if used properly, for these areas containing skewed markers may have higher recombination ratio for specific traits^40^.

In addition to the high recovery of type IIB restriction fragments of BcgI, RAD typing^41^ was used to genotype all dominant (presented in only one parent) and co-dominant (presented in both parents) markers. As more than half of 2b-RAD markers were made up of dominant markers^13^, using software only genotyping co-dominate markers like Stacks^42^ will greatly waste genetic information offered by sequencing reads. In this study, we constructed 2,219 (28.49%) dominant out of 7,786 reference markers. In the final 3,121 markers, 1,474 (47.22%) were dominant markers, and this proportion is similar to that in bivalve mollusc^13^. At the same time, high macro-collinearity among 2b-RAD markers of our map and the zebrafish genome indicated that the genotyping of dominant and co-dominant markers is free from error^32^.

Growth-related traits are economically and ecologically important traits and their genetic basis is thought to be highly polygenic in most organisms. Mapping QTL for growth-related traits is critically important in molecular breeding. Many QTL studies of growth-related traits have been conducted in common carp^43^,^44^, Asian seabass^45^, nine-spined stickleback^46^ and Atlantic salmon^47^. Only one study was carried out about the QTL of growth-related traits in a hybrid progenies between bighead carp and silver carp^48^. In this study, 5 morphometric body traits were measured and QTL responded for them were detected.

Here, we have found one significant QTL and 37 suggestive QTL for growth-related traits in bighead carp. For these QTL, we found most of their locations were overlapped on linkage groups (Supplementary Table S3). For example, marker ref-8593_27 locating on LG15 corresponded to five QTL, including qTL15-a, qBL15-b, qBH15-a, qHL15-a and qBW15-c. Meanwhile, each of seven locations on LG19, LG11, LG22, LG18, LG3 were corresponding to four QTL. For the rest of five QTL, three were overlapped in one genomic location and other two QTL were on two different genomic locations. One reason for this result might be the high correlation coefficients among the 5 traits in bighead carp. As can be seen from Table 2, the lowest correlation coefficients were 0.82 between BH and HL and the highest correlation coefficients were 0.98 between TL and BL. Based on this, we could conclude that there was a strong almost linear relationship among the 5 traits. And even further, QTL in one trait might be response for all other four traits. In bighead carp breeding, selection for one of the five traits may also result in improvement of others without use all measurement traits. Another reason for this result might be growth-related traits of bighead carp were controlled by only a few major areas in the genome. This pattern was similar to the pattern of QTL for growth-related traits in nine-spined stickleback^46^, common carp^44^ and chicken^49^. There are two patterns of growth-related QTL in fish, one is single major QTL accounted for significant portion of a trait and the other is many QTL with small or intermediate effects on the trait. Previous study compared growth rate in two pufferfish found that juvenile body size was mapped to only one QTL, which indicated single major gene mode of inheritance^50^. However, more studies uncovered that several QTL influenced growth in the Mexican cavefish^51^, the lake whitefish^52^ and nine-spined stickleback^46^. In this study, our results demonstrated that there are 4–9 QTL for each of the five traits, which indicated that bighead carp may be putative example of multiple QTL influencing on phenotypic variations of growth rate.

It has been reported that early growth in most tissues was caused by cell division, whereas in later stage growth was caused by an increase in cell size^53^. As the sex maturation for bighead carp can take 4–5 years, the sample time in this study (270 dpf) should be considered as early stage of the bighead carp lifespan. To study the functional background of these candidates QTL, we mapped all markers in QTL regions to bighead carp genome and searched their ortholog in zebrafish genome. For the QTL common to all five traits, we found it was surrounded by *CRP1* and *CRP2*, which are potent smooth muscle differentiation cofactors that may play roles in muscle cell division^54^. Besides, we also found a QTL common to four traits was involved in *SASH1*, which regulates cell proliferation and apoptosis in human^27^,^28^.

This study represents the first ultra-high density genetic map for bighead carp, which has 3,121 SNP markers in it. This map was constituted by 24 linkage groups and they showed good synteny information with zebrafish genome. The average marker spacing was 0.75 cM. Using this linkage group, we mapped 38 QTL for five growth related traits. Five out of these 38 QTL were located on the same location on LG15, and QTL on this place was significant on genome wide level for body length. According to the mapping result of the markers in LG15, we found it was surrounded by *CRP1* and *CRP2*, which played roles in muscle cell division. Markers flanking the QTL can be used to select fishes with better growth related traits.

## Methods

### Ethics statement

All experimental procedures involving the fish in this study were approved by the Committee for Animal Experiments of the Institute of Hydrobiology of the Chinese Academy of Sciences, China. The methods used in this study were carried out in accordance with the Laboratory Animal Management Principles of China. The rearing activities of bighead carp in Wuhan, Hubei were approved by the owner of the pond.

### Mapping families and phenotypic data

The fish formed the parental generation (F_0_) were collected from Zhangdu Lake (Wuhan; 30° 40′ 03″N, 114° 43′ 51″E) in 2009. The female fish was crossed *in vitro* with the male one in May, 2011 and the resulting F_1_ –offspring was group-reared in one mud pond. At the same time, the mud pond cultured grass carp (*Ctenopharyngodon idella*) and common carp (*Cyprinus carpio*). Although bighead carp mainly feed on filtering algae in the water, feed was put into the pond for the growth of common carp. A total of 117 full-sib F1 progeny were sampled to construct a linkage map. Growth related traits including body length (BL), body height (BH), body weight (BW), head length (HL) and total length (TL) were measured only once for all 117 offspring at 240 dph (days post hatch). This family exhibited high within family variation of growth related traits and therefore was used for linkage construction and QTL analysis.(Supplementary Table S4).

### 2b-RAD sequencing

Libraries for 2b-RAD were prepared for two parents and 117 progenies according to the protocol developed by Wang *et al*.^10^ but did some modifications (Supplementary Figure 1). Fin clips cut from all samples were preserved in 95% ethanol at room temperature for DNA samples. Genomic DNA was extracted following the standard phenol-chloroform protocol^55^. The concentration of extracted DNA was determined using a spectrophotometer. We used BcgI enzyme (NEB, UK) to digest genomic DNA of parents and offspring. The sequences cut by BcgI enzyme were ligated to adaptors with 5′- NN - 3′ overhangs. A unique six base pair barcode was used in each library during library preparation, and then all libraries were mixed together for Illumina single-end sequencing (1*50 bp). The sequencing was carried out by Shanghai Sangon Inc, Shanghai. To maximize the chances of discovering segregating SNPs, the parents were sequenced at a substantially higher depth than the progenies (Supplementary Table 2). All the 2b-RAD sequencing reads were deposited in the NCBI sequence read archive (SRA) database (accession no. SRP070967).

### Bioinformatics pipeline and SNP calling

The process for generating SNP genotype in this study was as follows. Firstly, raw reads were assigned to each sample according to their unique six base pair barcode. Secondly, raw reads were trimmed to remove adaptors and the terminal 2-bp positions were discarded to eliminate artifacts that might be arisen by ligation. Finally, reads contained no restriction site, long homo-polymer regions (>10 bp), ambiguous bases (N) or reads of low quality (>2 bp with quality less than Q20) were removed. The remaining high quality reads were used for further genotyping. Denovo genotyping was performed by RADtyping v1.3.0^41^ with default parameters. This software used stringent criteria in filtering candidate markers. It removed markers with too much coverage compared with the average depth of all markers. Only these loci with at least 6 reads and the minority allele had at least 2 reads supporting were kept in the following analysis.

### Linkage map construction and synteny analysis

Markers genotyped in at least 80% of all the samples were considered as high-quality markers and used for further analysis. Markers with significant segregation distortion (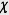
^2^ test, P < 0.001, df = 2) were excluded from the following map construction. Linkage map was created using JoinMap 4.0 software^56^. Linkage groups were identified with an independence logarithm of odds (LOD) threshold of 11.0. Unlinked markers and small linkage groups with less than 3 markers were not included from further analysis. The regression mapping algorithm was used for map construction. Map distances were estimated for each linkage group using Kosambi function. The map was visualized using MapChart ver.2.21.

To uncover the synteny information between bighead carp and zebrafish, we mapped all tags within 24 LGs to zebrafish genome to get their corresponding locus. As the type IIB restriction fragment was only 32 bp, it’s difficult to be used as query in searching zebrafish genome. Therefore, we firstly mapped the short sequences to bighead carp genome (Shunping He *et al*., unpublished data) and then we extracted 100 bp in each end flanking the mapping site of every uniquely mapped marker and used all 232 bp sequences as query to blast the zebrafish genome.

### Identification of segregation distortion region

After filtering these skewed markers with P < 0.001, we carefully check the position of markers with P < 0.01. If there are four consecutive skewed markers in one region, we defined this place as segregation distortion region. To uncover the genes within these regions, we mapped all skewed markers to bighead carp genome (Shunping He *et al*. unpublished data). Based on the position of all annotated genes and the skewed markers, we found genes located nearby these skewed markers. The position information about these skewed markers can be obtained upon request.

### QTL analysis

QTL mapping for growth traits was carried out with MapQTL version 6.0^57^. Multiple QTL Mapping (MQM) method was used to detect any significant associations between marker loci and growth-related traits. LOD statistics were calculated at an interval of 1 cM. The genome-wide (significance level) and linkage group-wide (suggestive level) LOD threshold were estimated using the permutation test (10,000 replicates) with a confidence interval of 95%. Graphic illustrations of the QTL profiles were generated using the program MapChart 2.2^58^.

## Additional Information

**How to cite this article**: Fu, B. *et al*. A high-density genetic map and growth related QTL mapping in bighead carp (*Hypophthalmichthys nobilis*). *Sci. Rep.* **6**, 28679; doi: 10.1038/srep28679 (2016).

## Supplementary Material

Supplementary Figure

Supplementary Table

## Figures and Tables

**Table 1:** Summary statistics of the sex-averaged linkage map of bighead carp.

LG Name	Num of SNPs	LG length(cM)	Average Dist(cM)	Largest Gap(cM)
LG1	109	123.841	1.136155963	6.636
LG2	76	77.742	1.022921053	9.164
LG3	174	107.931	0.620293103	4.835
LG4	80	68.534	0.856675	4.989
LG5	118	102.407	0.867855932	4.838
LG6	109	77.082	0.707174312	9.946
LG7	109	76.585	0.702614679	8.759
LG8	350	145.428	0.415508571	3.602
LG9	192	102.288	0.53275	4.768
LG10	77	92.693	1.203805195	5.343
LG11	103	114.211	1.10884466	5.19
LG12	170	90.881	0.534594118	4.955
LG13	151	97.681	0.64689404	12.86
LG14	95	89.481	0.941905263	6.064
LG15	105	101.486	0.966533333	5.801
LG16	121	109.233	0.902752066	14.552
LG17	103	92.183	0.894980583	5.323
LG18	103	91.019	0.883679612	4.093
LG19	92	85.724	0.931782609	3.09
LG20	93	82.143	0.883258065	8.631
LG21	150	119.297	0.795313333	3.203
LG22	240	109.188	0.45495	3.253
LG23	82	86.766	1.058121951	7.271
LG24	119	97.443	0.818848739	6.136
**TOTAL**	**3121**	**2341.26**	**0.75**	**14.552**

LG: linkage group; cM: centiMorgan.

**Table 2:** Pairwise correlation for all 5 growth-related traits in bighead carp.

	TL	BL	BH	HL	BW
TL	1				
BL	0.987885	1			
BH	0.883167	0.871764	1		
HL	0.911896	0.91138	0.82535	1	
BW	0.964491	0.968115	0.918774	0.872767	1

TL: total length. BL: body length. BH: body height. HL: head length. BW: body weight.

**Table 3:** All QTL for five growth-related traits.

Trait	QTL name	Marker	LG	Position(cM)	LOD	LOD Threshold		PVE(%)
						Genome-wide	Group-wide	
Total length	qTL15-a	ref-8593_27	15	23.97	8.28	8.4	3.9	30.2
	qTL11-b	ref-24929_7	11	52.318	7.33	8.4	4.6	27.3
	qTL11-c	ref-65692	11	55.227	6.95	8.4	4.6	26.1
	qTL11-d	ref-67070_2	11	96.315	6.86	8.4	4.6	25.8
	qTL19-a	ref-20240_31	19	6.099	6.71	8.4	4.1	25.3
	qTL18-a	ref-36051	18	51.107	5.87	8.4	4.5	22.5
	qTL22-a	ref-34298	22	54.484	5.52	8.4	4.3	21.3
	qTL3-b	ref-51859	3	27.12	5.09	8.4	4.3	19.8
	qTL3-a	ref-66486_3	3	4.741	4.64	8.4	4.3	18.3
Body length	qBL15-a	ref-8593_27	15	23.97	8.05	8	4	29.5
	qBL11-c	ref-65692	11	55.227	7.55	8	4.8	27.9
	qBL11-b	ref-24929_7	11	52.318	6.93	8	4.8	26
	qBL11-d	ref-67070_2	11	96.315	6.52	8	4.8	24.7
	qBL19-a	ref-20240_31	19	6.099	6.38	8	3.8	24.2
	qBL18-b	ref-36051	18	51.107	5.76	8	4.3	22.1
	qBL22-a	ref-34298	22	54.484	5.23	8	4.2	20.3
	qBL3-b	ref-51859	3	27.12	5.07	8	4.2	19.8
	qBL3-a	ref-66486_3	3	4.741	4.61	8	4.2	18.2
Body heigth	qBH22-a	ref-34298	22	54.484	6.07	7.4	4	23.2
	qBH11-b	ref-65692	11	55.227	5.96	7.4	4.2	22.8
	qBH11-a	ref-24929_7	11	52.318	5.76	7.4	4.2	22.2
	qBH15-a	ref-8593_27	15	23.97	5.23	7.4	3.9	20.3
	qBH3-b	ref-15932_2	3	60.679	4.6	7.4	4.1	18.1
	qBH3-a	ref-51859	3	27.12	4.48	7.4	4.1	17.7
	qBH18-a	ref-36051	18	51.107	4.26	7.4	4	16.9
	qBH11-c	ref-67070_2	11	96.315	4.22	7.4	4.2	16.7
	qBH19-a	ref-20240_31	19	6.099	3.84	7.4	3.7	15.4
Head length	qHL15-a	ref-8593_27	15	23.97	11.08	12.3	4.1	38.2
	qHL22-a	ref-34298	22	54.484	5.96	12.3	4.7	22.8
	qHL3-b	ref-51859	3	27.12	5.51	12.3	4.6	21.3
	qHL3-a	ref-66486_3	3	4.741	4.96	12.3	4.6	19.4
Body	qBW11-b	ref-65692	11	55.227	5.75	5.8	4	22.1
weight	qBW11-a	ref-24929_7	11	52.318	5.39	5.8	4	20.9
	qBW15-a	ref-8593_27	15	23.97	5.19	5.8	3.7	20.2
	qBW11-c	ref-67070_2	11	96.315	4.88	5.8	4	19.1
	qBW15-b	ref-4159	15	70.05	4.59	5.8	3.7	18.1
	qBW18-a	ref-36051	18	51.107	4.51	5.8	3.8	17.8
	qBW19-a	ref-20240_31	19	6.099	4.42	5.8	3.7	17.5

PVE: phenotypic variance explained.

**Table 4:** Comparison of genome feature for three bighead carp genetic linkage maps.

	This study	Liao *et al*.^22^	Zhu *et al*.^32^
Number of linkage groups recovered	24	30	24
Genome size estimate	2379.5	1209.0	2067.1
Genome coverage estimate	98.3%	82.8%	92.8%
Number of markers used for map	3121 SNPs	153(27 SSRs & 126 AFLPs)	659 SSRs
Map length [cM]	2341.3	852.0	1917.3
Average length of marker interval	0.75	7.0	2.9
Linkage group size range [cM]	68.5–145.4	2.2–74.9	52.2–133.5
