# Supplementary material for: A high-density genetic map and growth related QTL mapping in bighead carp (Hypophthalmichthys nobilis)
Source: Sci Rep. 2016 Jun 27;6:28679. doi: 10.1038/srep28679 (PMC4921863; doi:10.1038/srep28679)

A high-density genetic map and growth related QTL mapping in bighead carp (*Hypophthalmichthys nobilis*)

Beide Fu[1], Haiyang Liu[1,2], Xiaomu Yu[1], Jingou Tong[1,*]

[1]State Key Laboratory of Freshwater Ecology and Biotechnology, Institute of Hydrobiology, Chinese Academy of Sciences, Wuhan 430072,China

[2]University of Chinese Academy of Sciences, Beijing 100039, China

Supplementary Fig. S1. Pipeline for a 2b-RAD sequencing library construction.

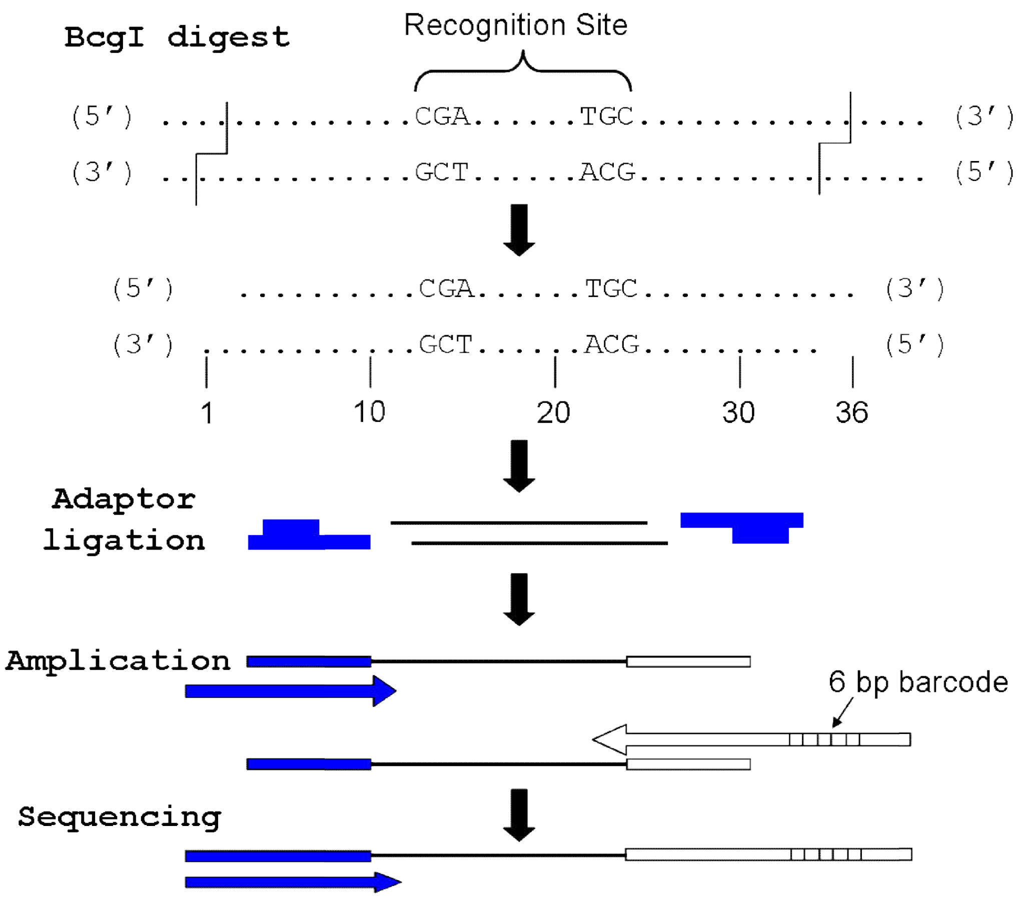

Supplementary Fig. S2 Number of markers for each segregation pattern.

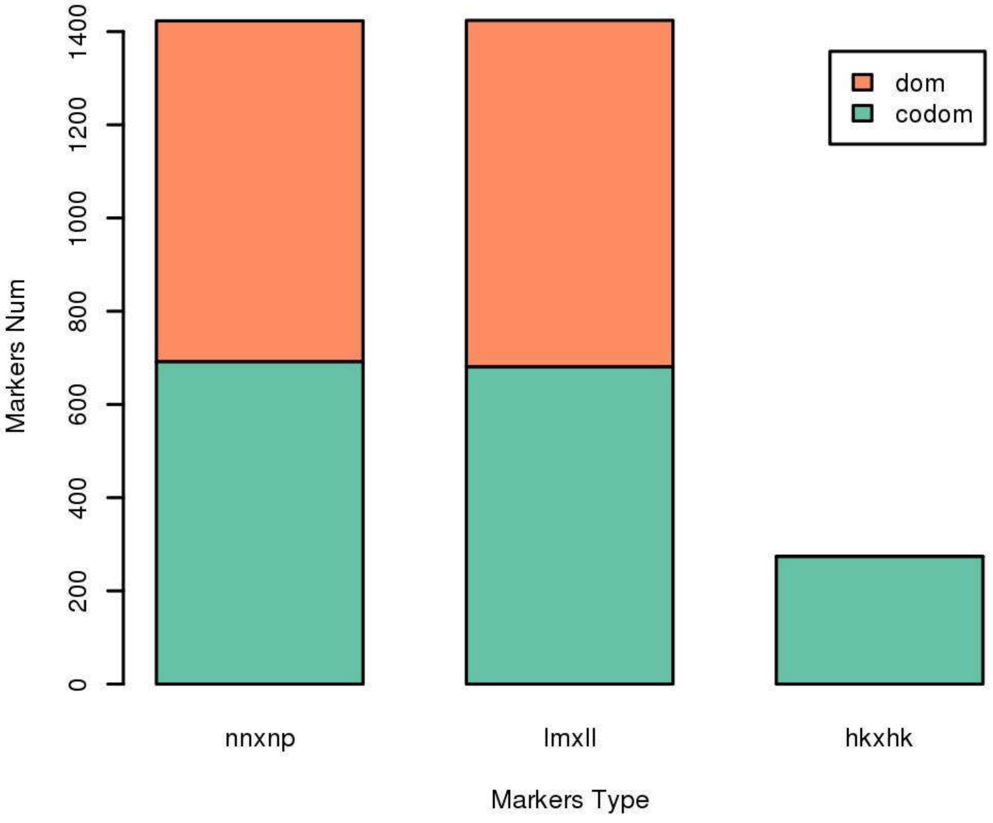

Supplement: Supplementary Figure [file srep28679-s1.pdf]
